# Geography, niches, and transportation influence bovine respiratory microbiome and health

**DOI:** 10.3389/fcimb.2022.961644

**Published:** 2022-09-12

**Authors:** Jianmin Chai, Xinting Liu, Hunter Usdrowski, Feilong Deng, Ying Li, Jiangchao Zhao

**Affiliations:** ^1^ Guangdong Provincial Key Laboratory of Animal Molecular Design and Precise Breeding, College of Life Science and Engineering, Foshan University, Foshan, China; ^2^ School of Life Science and Engineering, Foshan University, Foshan, China; ^3^ Division of Agriculture, Department of Animal Science, University of Arkansas, Fayetteville, AR, United States

**Keywords:** respiratory microbiota, bovine, metagenomics, geography, bovine respiratory disease, transportation, nasopharynx, lung

## Abstract

Bovine respiratory disease (BRD), one of the most common and infectious diseases in the beef industry, is associated with the respiratory microbiome and stressors of transportation. The impacts of the bovine respiratory microbiota on health and disease across different geographic locations and sampling niches are poorly understood, resulting in difficult identification of BRD causes. In this study, we explored the effects of geography and niches on the bovine respiratory microbiome and its function by re-analyzing published metagenomic datasets and estimated the main opportunistic pathogens that changed after transportation. The results showed that diversity, composition, structure, and function of the bovine nasopharyngeal microbiota were different across three worldwide geographic locations. The lung microbiota also showed distinct microbial composition and function compared with nasopharyngeal communities from different locations. Although different signature microbiota for each geographic location were identified, a module with co-occurrence of *Mycoplasma* species was observed in all bovine respiratory communities regardless of geography. Moreover, transportation, especially long-distance shipping, could increase the relative abundance of BRD-associated pathogens. Lung microbiota from BRD calves shaped clusters dominated with different pathogens. In summary, geography, sampling niches, and transportation are important factors impacting the bovine respiratory microbiome and disease, and clusters of lung microbiota by different bacterial species may explain BRD pathogenesis, suggesting the importance of a deeper understanding of bovine respiratory microbiota in health.

## Introduction

Bovine respiratory disease (BRD) is one of the most common infectious causes of pneumonia in cattle worldwide and causes morbidity and mortality in newly transported and recently weaned feedlot cattle, resulting in a slower growth rate and economic cost for prevention and treatment (Chai et al., 2022). With developments of sequencing technology, the roles of the respiratory microbiome in maintaining homeostasis of the airway ecosystem and its association with disease are more deeply understood (Dickson et al., 2016; Man et al., 2017; Zeineldin et al., 2019). The most implicated bacterial pathogens in BRD, including *Mycoplasma bovis*, *Mannheimia haemolytica*, *Histophilus somni*, and *Pasteurella multocida*, have been identified (Nicola et al., 2017; Cirone et al., 2019). Until now, 16S rRNA sequencing has been the most popular technology to investigate the respiratory microbiome of both humans and cattle (Human Microbiome Project, 2012; Zeineldin et al., 2020). Metagenomics that capture sequences from nearly all the organisms inhabiting the respiratory system is more effective and accurate to investigate the composition and functions of the respiratory microbiome (Holman et al., 2017c; Cui et al., 2021). However, fewer studies regarding BRD using metagenomics have been performed.

Geography serves as one of the major factors influencing the respiratory microbiota (Gupta et al., 2017). As cattle are a main source of human protein and nutrition and BRD appears worldwide in cattle, research of the bovine respiratory microbiome at the world level is important and necessary. However, due to feeding strategy, diet, environment, climate, *etc*., the compositional differences of the bovine airway microbiota are affected by the geographic locations of farms. Using 16S sequencing technology, a study found that, in Canada, the most prominently identified genera in the nasopharynx of calves were *Mycoplasma*, *Lactococcus*, *Moraxella*, *Histophilus*, and *Pasteurella* (Mcmullen et al., 2018), while in the United States *Mannheimia*, *Mycoplasma*, *Moraxella*, *Psychrobacter*, and *Pseudomonas* were the top five genera in the nasopharynx of calves (Lima et al., 2016). However, to our knowledge, there are fewer studies to specifically investigate how bovine respiratory microbiota and its functions vary geographically.

Other factors, including sampling niche in the respiratory tract, weaning and transportation, time since arrival to the feedlot, and health status, can influence the microbial structure of the bovine respiratory ecosystem and are associated with an increased risk of BRD in recently weaned beef calves (Snowder et al., 2006; Taylor et al., 2010; Zeineldin et al., 2017a; Zeineldin et al., 2020). The biochemical and physiological environments of niches within the respiratory tracts are different, resulting in a different microbial composition (Zeineldin et al., 2019; Chai et al., 2022). A recent study reported that the dominant bacteria were *Moraxella* and *Mycoplasma* in the nasopharynx, as well as *Mycoplasma* in the lungs of healthy calves, and concluded that the nasopharynx could serve as a primary source for lung microbiota (Mcmullen et al., 2020). Although a recent theory that suggests microbiota colonize in the upper respiratory tracts firstly and then disperse to the lungs was reported in humans (Venkataraman et al., 2015), it has not been confirmed in cattle. Furthermore, the nasopharynx has been the most popular niche to determine the relationship between the respiratory microbiota and BRD (Holman et al., 2015a; Timsit et al., 2016a; Holman et al., 2017a; Zeineldin et al., 2017a; Holman et al., 2017d)—for instance, some studies mainly focused on the pathogens in the nasopharynx (Timsit et al., 2016b; Gaeta et al., 2017; Zeineldin et al., 2017a; Holman et al., 2017d; Holman et al., 2019; Mcmullen et al., 2019). In addition, transportation, as an important stressor, is associated with the bovine respiratory microbiota and BRD (Holman et al., 2017d; Mcmullen et al., 2018; Cui et al., 2021). There have been no studies, however, investigating whether consistent microbial changes by stressors (such as transportation) were found in calves from different geographic locations.

Three studies performed metagenomics to determine how transportation or BRD affects the nasopharyngeal microbiome of calves from Canada (Malmuthuge et al., 2021) and China (Cui et al., 2021) and the lung microbiota of calves from Canada (Klima et al., 2019). Their studies provide experimental data that can be analyzed to determine the effects of geography and transportation stress on bovine microbial structure and functional potential. This paper presents the results of our re-analysis of the shotgun metagenomic dataset from these three studies to determine the geographic and transportation effects on the bovine respiratory microbiome.

## Materials and methods

### Data collection

Our study was based on three public metagenomic datasets published by Malmuthuge et al. (2021); Cui et al. (2021), and Klima et al. (2019). Malmuthuge *et al*. investigated the effects of weaning and transportation on the temporal dynamics of the nasopharyngeal microbiota in Hereford-crossed calves in Canada, Cui and colleagues measured the longitudinal changes of the nasopharyngeal microbiome before and after long-distance transportation in Simmental calves in China, and Klima *et al*. collected and characterized the lung microbiota from feedlot calves that died from BRD (**Supplementary Table S1**). A total of 145 bovine respiratory samples collected from the feedlot calves 5 to 6 months of age were included in this study. In brief, the samples were from three geographic locations [Saskatoon in Canada, two cities (Qiqihaer and Guangan) in China, and Alberta in Canada] and two niches (nasopharynx and lung). Moreover, transportation effects were estimated in the studies of Malmuthuge *et al*. and Cui *et al*. Details of the study design and sample collections were described in the original study. Metagenomic sequencing was performed using the Illumina HiSeq platform. Sequences were downloaded from the NCBI SRA database under accession codes PRJNA687519, PRJNA724913, and PRJNA395911.

### Metagenomic sequence processing

KneadData (v 0.7.2) was used to remove host contamination and filter reads for the downloaded metagenomic data. Low-quality reads with a Phred score smaller than 15 within a 5-bp sliding window on reads were trimmed using Trimmomatic (v0.39). Clean reads of each sample were acquired, and then all reads were aligned to the *Bos taurus* reference genome (UMD v3.0) using bmtagger (v.3.102.4) for host contamination read removal. Next, clean reads of each sample were then uploaded into the MG-RAST metagenomic analysis server (v4.0) (Keegan et al., 2016) and against the Kyoto Encyclopedia of Genes and Genomes (KEGG) Orthology database for function analysis and the RefSeq database for microbial taxa classification based on the recommended manual. After obtaining the count table of microbial composition and functional prediction from the MG-RAST server, R software was used to do downstream analyses.

### Statistics and bioinformatics

Alpha diversity, including the Shannon index and richness, was calculated to evaluate the corresponding diversities by R (v4.1.2). A two-tailed Wilcoxon signed-rank test was used to test the significance of alpha diversity. Principal coordinate analysis based on Bray–Curtis distance was performed to visualize beta diversity, and dissimilarity among groups was determined using the “adonis2” function on the R “vegan” package. Signature microbiota for geographic locations were identified using the linear discriminant analysis (LDA) effect size (LEfSe) with default settings (*e*.*g*., LDA score >2 as a criterion for judging the significant effect size), and the abundances of microbial signatures were visualized using a heat map. The Sankey plot was drawn in R to show the taxonomy of bovine respiratory microbiome in three geographic locations. Other boxplots were made using the “ggplot2” package in R.

To determine the correlation of the bovine respiratory microbiome in the network interface, correlation matrixes by calculating pairwise Spearman’s rank correlations among the top 200 bacteria were constructed. A correlation between two bacteria was considered statistically robust if Spearman’s correlation coefficient (*R*) was greater than 0.5, and the *P*-value was less than 0.01. To avoid false positives, all *P*-values were adjusted with a multiple testing correction using the Benjamini–Hochberg method. The pairwise correlations of the bacteria formed their co-occurrence networks. Network analyses were performed using “VEGAN”, “igraph”, and “Hmisc” packages in R, and the interactive platform of Gephi was employed for network visualization. The size of each node is proportional to the number of connections.

Hierarchical clustering based on the relative abundance of the bronchoalveolar lavage (BAL) microbiome was performed using the Bray–Curtis distance and the “hclust” function in R. Taxa of the top 30 lung microbiota were included in hierarchical clustering. All BAL samples were subjected to unsupervised hierarchical clustering.

## Results

### Sample characteristics and sequencing analysis

Metagenomic samples (*n* = 145) during the feedlot period were obtained from three worldwide studies with publicly available datasets (Klima et al., 2019; Cui et al., 2021; Malmuthuge et al., 2021). The characteristics of the samples are summarized in **Supplementary Table S1**. Three geographic locations, including Saskatoon in Canada, two cities (Qiqihaer and Guangan) in China, and Alberta in Canada, were selected. The respiratory microbial samples were collected from the nasopharynx and lung using nasopharyngeal swabs (NPS; *n* = 130) and bronchoalveolar lavage (BAL; *n* = 15). Notably, the effects of short- and long-distance transportation, respectively, were included in the NPS, which were analyzed to estimate the shipping stress on the nasopharyngeal microbiota. After quality control, an average of 3,374,490 clean reads in the respiratory metagenomics were used for downstream analysis, including microbial classification using the RefSeq database and functional prediction by the KEGG Orthology database.

### Bovine respiratory microbiome influenced by geographic locations and sampling niches

**Figure 1:**
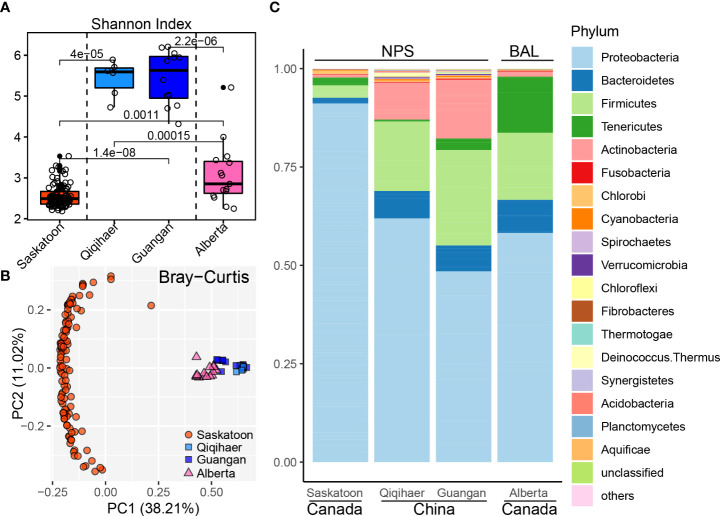
Bovine respiratory microbiome influenced by geographic locations and sampling niches. **(A)** Shannon index of bovine respiratory microbiota among NPS of Saskatoon (Canada), NPS of Qiqihaer and Guangan (China), and BAL of Alberta (Canada). Alpha diversity was tested using the Kruskal–Wallis test. The line inside the box denotes the median, and the boxes denote the interquartile range between the first and third quartiles (25th and 75th percentiles, respectively). **(B)** Principal coordinate analysis of the Bray–Curtis distances between microbiota. **(C)** Bacterial abundances at the phylum level. NPS, nasopharyngeal swab; BAL, bronchoalveolar lavage.

To analyze the effects of geographic location and sampling niches on the bovine respiratory microbiome, microbial diversities were estimated at the community level. The Shannon index of NPS of Qiqihaer and Guangan was higher compared with the NPS of Saskatoon and the BAL of Alberta (*P* < 0.05) (**Figure 1A**). Interestingly, the BAL of Alberta had greater alpha diversity than the NPS of Saskatoon (*P* < 0.05). Consistently, the NPS of Qiqihaer and Guangan showed distinct clustering compared with the NPS of Saskatoon (analysis of similarity, ANOSIM: *R* = 0.99, *R* = 1.0, *p* = 0.001) and the BAL of Alberta (ANOSIM: *R* = 0.78, *R* = 0.49, *p* = 0.001) based on the Bray–Curtis distance (**Figure 1B**). At the same time, significant differences between Saskatoon and Alberta (ANOSIM: *R* = 0.99, *p* = 0.001) were also observed.

Using the Refseq database, 96.72% high-quality reads were classified as the bacterial kingdom, indicating the bacteria as the main microbiome in the bovine respiratory tracts (**Supplementary Figure S1**). At the phylum level, a total of 27 bacterial phyla were identified across 145 bovine respiratory samples. The predominant phylum in the NPS of Saskatoon was Proteobacteria (91.19%). In the NPS niche of Qiqihaer and Guangan (China), the most dominant phyla were Proteobacteria (48.49 and 61.94%), Firmicutes (24.26 and 17.68%), Actinobacteria (14.90 and 9.40%), and Bacteroidetes (6.59 and 6.98%) (**Figure 1C**). The BAL of Alberta was dominated by Proteobacteria (58.27%), Firmicutes (17.03%), Tenericutes (14.27%), and Bacteroidetes (8.41%). At the genus level, *Burkholderia* (81.71%) was the dominant genus in the NPS of Saskatoon (**Supplementary Figure S2**). The top genera in the NPS of Qiqihaer and Guangan were *Psychrobacter* (8.07 and 11.63%), *Moraxella* (4.94 and 7.13%), and *Corynebacterium* (4.63 and 2.08%). In the BAL microbiome, the top genera included *Mannheimia* (16.55%), *Mycoplasma* (14.11%), *Actinobacillus* (10.51%), *Clostridium* (5.73%), *Psychrobacter* (5.66%), and *Haemophilus* (5.60%).

### The signature microbiota associated with geographic locations and sampling niches

**Figure 2:**
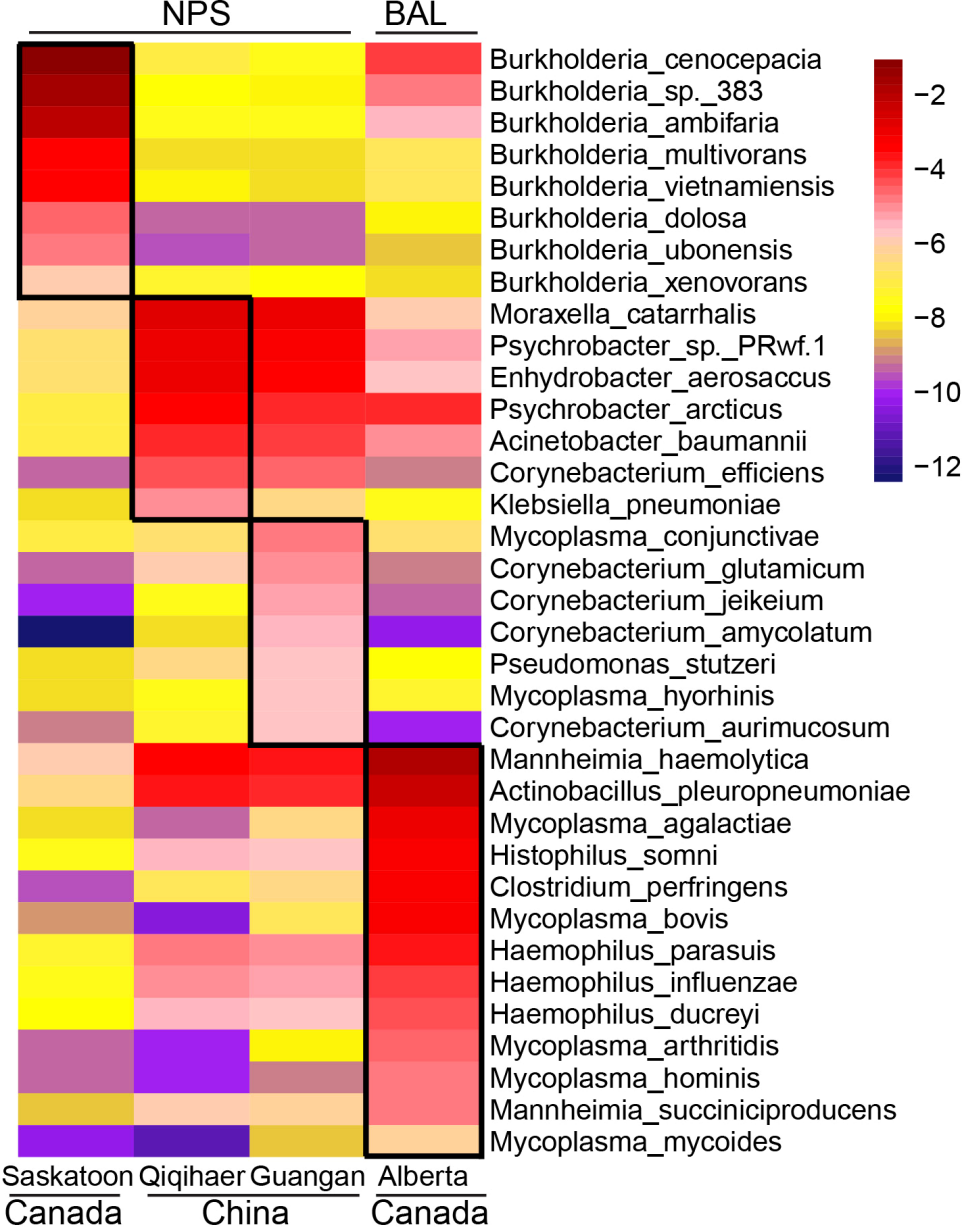
Geographic and niche-associated bacterial signatures that were identified by LEfSe analysis. The heat map shows the average relative abundance of ages on a log scale. The log-scaled relative abundance heat map of city-related bacterial species screened by LEfSe (linear discriminant analysis >2) in the bovine respiratory microbiome. The color of cells from purple to red corresponds to the relative abundance of bacteria from low to high. NPS, nasopharyngeal swab; BAL, bronchoalveolar lavage.

LEfSe was performed to identify the microbial species differentiating both cities and sampling niches. *Burkholderia* species, including *Burkholderia cenocepacia*, *Burkholderia* sp. *383*, *Burkholderia ambifaria*, *Burkholderia multivorans*, *Burkholderia vietnamiensis*, *Burkholderia xenovorans*, *Burkholderia dolosa*, *Burkholderia ubonensis*, and *Burkholderia phymatum*, were enriched in the NPS of Saskatoon (**Figure 2**), while the NPS of Qiqihaer and Guangan had greater abundances of bacteria such as *Moraxella catarrhalis*, *Psychrobacter* sp. *PRwf-1*, *Enhydrobacter aerosaccus*, *Psychrobacter arcticus*, *Corynebacterium efficiens*, *Corynebacterium glutamicum*, and *Mycoplasma conjunctivae*. In the BAL of Alberta (Canada), the abundant bacteria identified by LefSe were *Mannheimia haemolytica*, *Mannheimia succiniciproducens*, *Mycoplasma* species (*Mycoplasma agalactiae*, *Mycoplasma arthritidis*, *Mycoplasma bovis*, *Mycoplasma hominis*, and *Mycoplasma mycoides*), *Histophilus somni*, *Haemophilus* species (*Haemophilus ducreyi*, *Haemophilus influenzae*, and *Haemophilus parasuis*), *Actinobacillus* species (*Actinobacillus pleuropneumoniae* and *Actinobacillus succinogenes*), *Clostridium perfringens*, and *Prevotella* species (*Prevotella melaninogenica*, *Prevotella copri*, *Prevotella ruminicola*, *Prevotella buccae*, *Prevotella oris*, and *Prevotella bryantii*).

**Figure 3:**
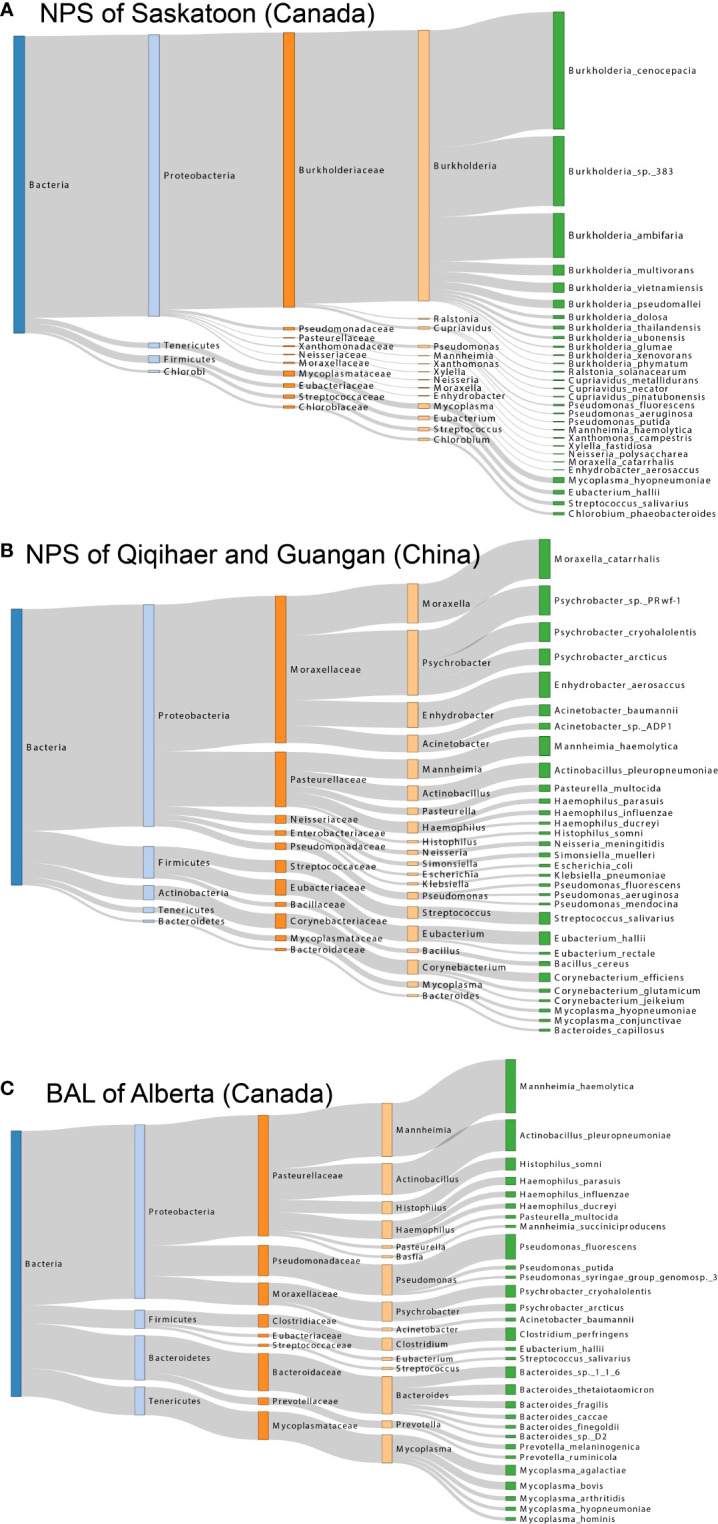
Sankey diagram for the major taxonomy structure of bovine respiratory microbiome. **(A**, **B**, and **C)** Sankey diagrams of bovine airway microbiota among NPS of Saskatoon (Canada), NPS of Qiqihaer and Guangan (China), and BAL of Alberta (Canada), which depict the bacterial flow of taxonomy from the kingdom to species level. NPS, nasopharyngeal swab; BAL, bronchoalveolar lavage.

To better visualize the microbial composition of the bovine respiratory microbiome among geographic locations, a Sankey diagram of bacterial taxonomy was drawn (**Figure 3**). The most abundant bacteria in the NPS of Saskatoon were *Burkholderia* species, including *B. cenocepacia*, *B.* sp. *383*, and *B. ambifaria* (*Burkholderiaceae* family, Proteobacteria phylum) (**Figure 3A**). The top bacteria in the NPS of Qiqihaer and Guangan were species under the *Moraxellaceae* family (*Moraxella catarrhalis*, *Psychrobacter* sp. *PRwf-1*, and *Enhydrobacter aerosaccus*) and the *Pasteurellaceae* family (*Mannheimia haemolytica* and *Actinobacillus pleuropneumoniae)* belonging to the Proteobacteria phylum, followed by the *Streptococcaceae* family (*Streptococcus salivarius*) and the *Eubacteriaceae* family (*Eubacterium hallii*) belonging to the Firmicutes phylum (**Figure 3B**). In the BAL of Alberta (Canada), species members of the *Pasteurellaceae* family (*e*.*g*., *Mannheimia haemolytica*, *Actinobacillus pleuropneumoniae*, and *Histophilus somni*), the *Pseudomonadaceae* family (*e*.*g*., *Pseudomonas fluorescens*), and the *Moraxellaceae* family (*e*.*g*., *Psychrobacter cryohalolentis*) belonging to the Proteobacteria phylum, the *Clostridiaceae* family (*e*.*g*., *Clostridium perfringens*) belonging to the Firmicutes phylum, the *Bacteroidaceae* family (*e*.*g*., *Bacteroides* sp. *1_1_6* and *Bacteroides thetaiotaomicron*) belonging to the Bacteroidetes phylum, and the *Mycoplasmataceae* family (*e*.*g*., *Mycoplasma bovis* and *Mycoplasma agalactiae*) belonging to the Tenericutes phylum formed the major bacterial composition (**Figure 3C**).

### Co-occurrence analysis of bacterial interconnections within geographic locations and niches

**Figure 4:**
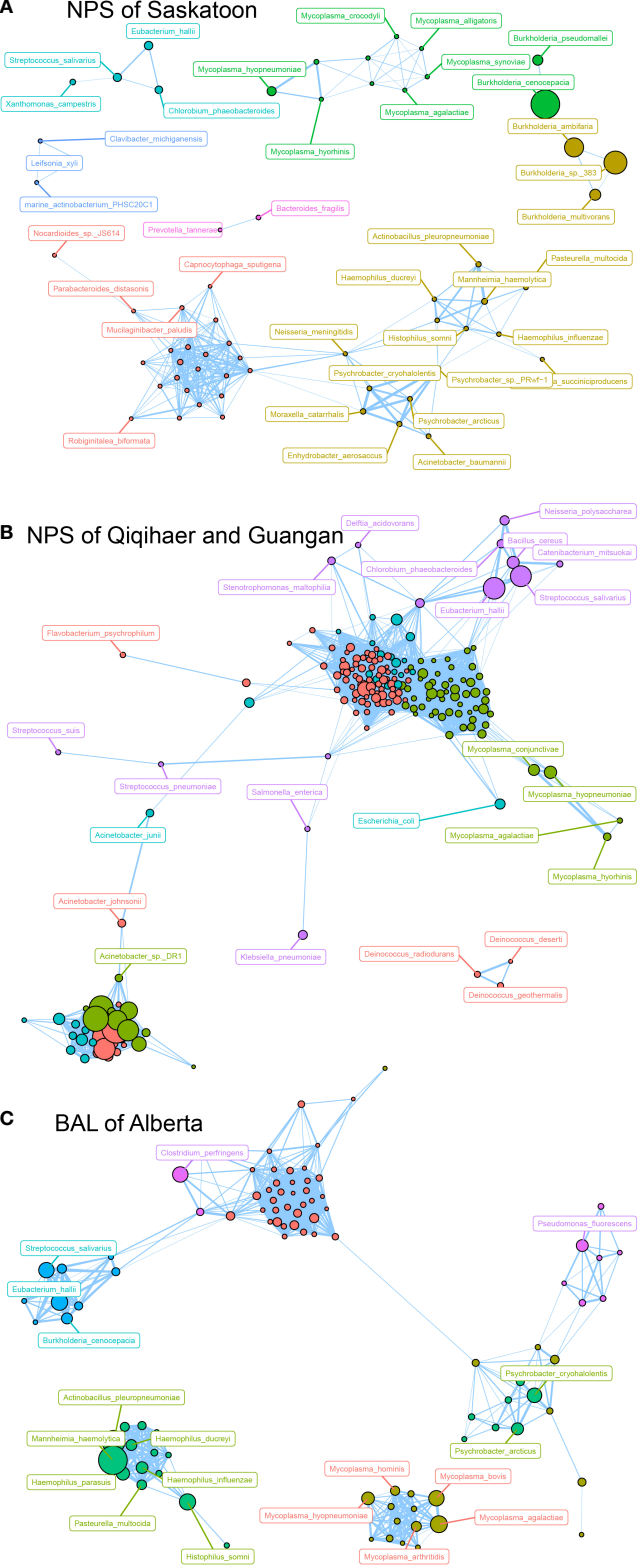
Network analysis revealing the co-occurrence patterns among airway microbial species. The nodes were colored according to modularity class. **(A–C)** Network analysis of bovine airway microbial species in samples of NPS of Saskatoon (Canada), NPS of Qiqihaer and Guangan (China), and BAL of Alberta (Canada). A connection represents a strong (Spearman’s correlation coefficient *R* > 0.8) and significant (*P* < 0.01) correlation. The size of each node is proportional to the number of connections, that is, the degree. NPS, nasopharyngeal swab; BAL, bronchoalveolar lavage.

Based on the modularity class, the entire network in the NPS of Saskatoon can be parsed into six major modules (**Figure 4A**). *Burkholderia* species, including *B. cenocepacia*, *B.* sp. *383*, *B. ambifaria*, *B. multivorans* and *B. pseudomallei*, formed a module, while *Mycoplasma* species (*M. agalactiae*, *Mycoplasma synoviae*, *Mycoplasma alligatoris*, *Mycoplasma crocodyli*, *Mycoplasma hyopneumoniae*, and *Mycoplasma hyorhinis*) co-occurred in another module. Regarding the NPS of Qiqihaer and Guangan (China), six modules with more complex bacterial co-occurrences were found. Interestingly, *Mycoplasma* species (*M. conjunctivae*, *M. hyopneumoniae*, *M. hyorhinis*, and *M. agalactiae*) also co-occurred (**Figure 4B**). In the BAL of Alberta community, *Mycoplasma* species (*M. agalactiae*, *M. bovis*, *M. arthritidis*, *M. hyopneumoniae*, and *M. hominis*), which were inter-connected with each other, were also observed (**Figure 4C**). Moreover, *Mannheimia haemolytica*, *Histophilus somni*, *Pasteurella multocida*, and *Actinobacillus pleuropneumoniae* were correlated with *Haemophilus* species (*H. ducreyi*, *H. influenzae*, and *H. parasuis*).

### Geographic locations and sampling niches affect the functions of the bovine respiratory microbiome

**Figure 5:**
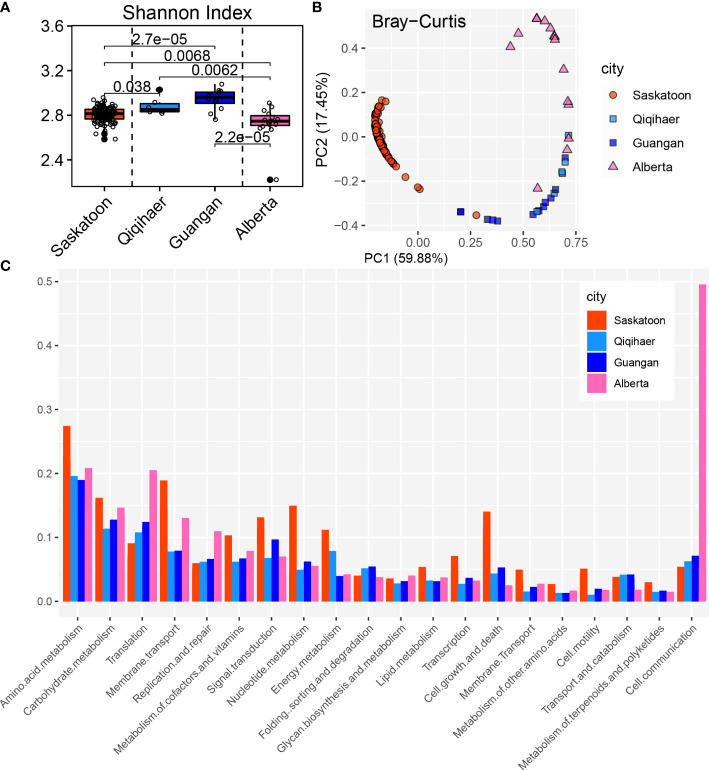
Functional analysis of bovine respiratory microbiome. **(A)** Shannon index of Kyoto Encyclopedia of Genes and Genomes (KEGG) function. **(B)** Principal coordinate analysis of the Bray–Curtis distance of the KEGG pathways. NPS, nasopharyngeal swab; BAL, bronchoalveolar lavage. **(C)** KEGG annotation at level 2.

Consistent with the bovine microbial structure, the KEGG functional configuration showed significant differences among cities and niches (**Figures 5A, B**). The Shannon index in the NPS of Qiqihaer and Guangan was higher compared with the NPS of Saskatoon and the BAL of Alberta (*P* < 0.05) (**Figure 5A**) and had distinct clustering compared with the NPS of Saskatoon (ANOSIM: *R* = 0.99, *R* = 0.98, *p* = 0.001) and the BAL of Alberta (ANOSIM: *R* = 0.30, *R* = 0.66, *p* = 0.001) based on the Bray–Curtis distance (**Figure 5B**).

The NPS of Saskatoon (Canada), the NPS of Qiqihaer and Guangan, and the BAL of Alberta had relative abundances of “cellular processes” (12.00, 8.92, 8.30, and 5.02%), “environmental information processing” (21.24, 14.99, 14.15, and 17.11%), and “genetic information processing” (11.79, 22.49, 22.16, and 23.28%) at level 1 (**Supplementary Figure S3**). At level 2, the NPS of Saskatoon had higher relative abundance of “membrane transport” (11.92%), “signal transduction” (6.60%), “transcription” (2.41%), “cell growth and death” (6.45%), “cell motility” (2.62%), “amino acid metabolism” (18.57%), “carbohydrate metabolism” (16.53%), “metabolism of cofactors and vitamins” (4.98%), “nucleotide metabolism” (3.06%), and “energy metabolism” (3.22%) (**Figure 5C**). The NPS of Qiqihaer and Guangan was greater in “folding, sorting, and degradation” (3.98%) and “signal transduction” (5.95%). The BAL of Alberta had higher relative abundances of “translation” (22.34%), “carbohydrate metabolism” (13.53%), “membrane transport” (10.34%), “replication and repair” (6.84%), and “cell communication” (5.51%).

### Long-distance transportation changes bovine nasopharyngeal microbiota

**Figure 6:**
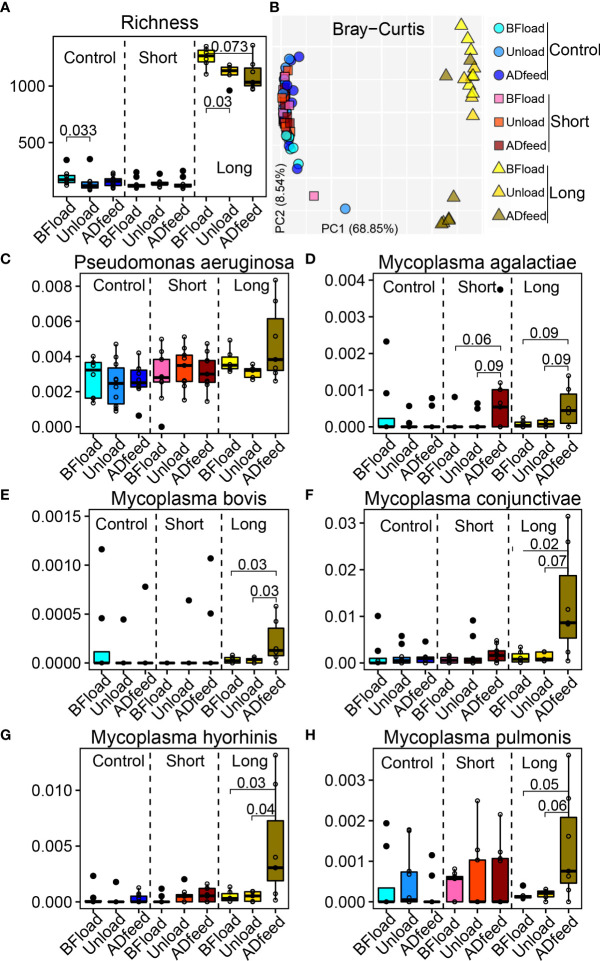
Long-distance transportation changes the bovine nasopharyngeal microbiota. **(A)** Richness. **(B)** Principal coordinate analysis plot based on the Bray–Curtis distance. **(C–H)** Main species associated with transportation. Control, non-transportation; Short, short-distance transportation; Long, long-distance transportation; BFload, time before loading to truck; Unload, unloading; ADfeed, 7 days after placement and adaptive feeding.

Three groups including non-transportation (Control) and short-distance (Short) and long-distance transportation (Long) were selected. Three days was chosen as the long-distance transportation from a study of Cui et al. (2021), and metagenomic data in the Long group at the time before loading to truck (Bfload), unloading (Unload), and 7 days after placement and adaptive feeding (Adfeed) were screened. Correspondingly, we selected data at the same time points from two other groups in the study of Malmuthuge et al. (2021). Interestingly, richness in the Long group at Unload and Adfeed time points significantly decreased (*P* < 0.05) compared with that at Bfload, while there were no temporal changes of alpha diversity in the Control and Short groups. Beta diversity based on the Bray–Curtis distance had consistent results (**Figures 6A, B**).

Next, LefSe was used to identify the longitudinal changes of microbiota following transportation (**Supplementary Figure S4**). Due to the variation from different studies and across multiple cities, the signature microbiota after transportation among the three groups were different. Then, we focused on the bacteria among studies to test if they have patterns across transportation distance and time. Although the microbial data were from different studies, shared bacteria, such as *Pseudomonas aeruginosa*, were not influenced by either short or long transportation, while *Mycoplasma agalactiae* was influenced by both short- and long-distance transportations such as higher abundances at Adfeed time in the Short and Long groups (**Figures 6C, D**). Specifically, some bacteria associated with BRD pathogens were influenced by long-distance transportation, such as the greater abundances of *Mycoplasma bovis*, *Mycoplasma conjunctivae*, *Mycoplasma pulmonis*, and *Mycoplasma hyorhinis* at the ADfeed of the Long group compared with at the BFload and Unload time points (**Figures 6E–H**).

### Colonization of the opportunistic pathogens at the nasopharynx changes with time following weaning and transportation

Measurement of the temporal dynamics of the bovine respiratory microbiome affected by stressors including weaning and short-distance transportation allows us to better understand the changes of the bovine respiratory microbiome at the feedlot, leading to a better understanding of BRD pathogenesis (Chai et al., 2022). Metagenomics of the bovine respiratory microbiome in treatment groups on days 0 (prior to weaning and short-distance transportation, WT), 2, 4, 8, 14, and 28 was used to compare with the control group that stayed with their dams (suckling). Slight differences between suckling and WT were found based on alpha and beta diversities (**Supplementary Figure S5**), while the temporal dynamics of the bovine respiratory microbiome changed significantly as more statistically significant differences were found among time points (**Supplementary Figure S6**).

**Figure 7:**
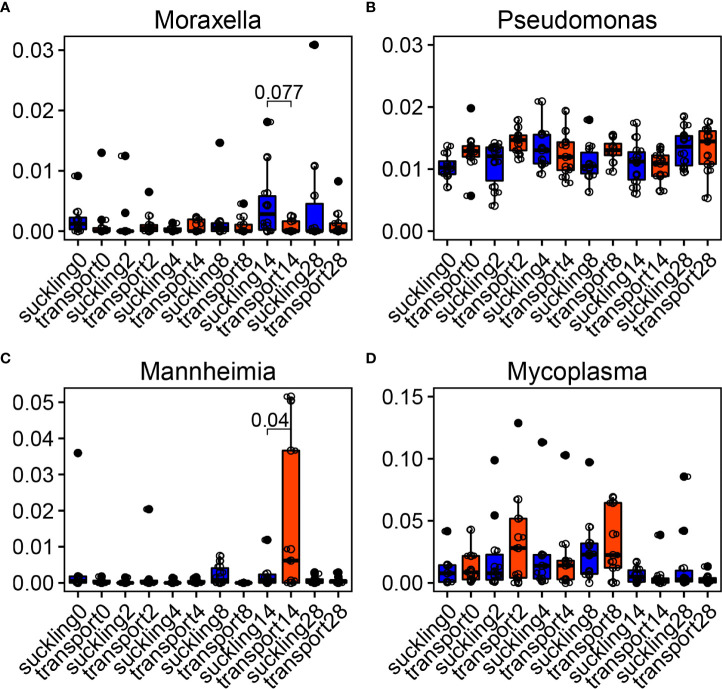
Temporal dynamics of bovine respiratory microbiota after weaning + transportation (WT). **(A–D)** Main bacterial genera changed after WT compared with the control group that stayed with their dams (suckling). Significances were labeled between suckling and WT at the time when they were statistically different.

The major bacterial genera associated with BRD were influenced by time and WT (**Figure 7**). *Moraxella* was more abundant in the suckling group on day 14 (**Figure 7A**). Another genus, *Pseudomonas*, was not influenced by WT since no differences were observed between the two groups during the trial (**Figure 7B**). *Mannheimia* was greater in WT at day 14, and *Mycoplasma* had higher medians than suckling, although there were no statistically significant differences (**Figures 7C, D**).

### Individual variances and clusters of the bovine lung microbiome

**Figure 8:**
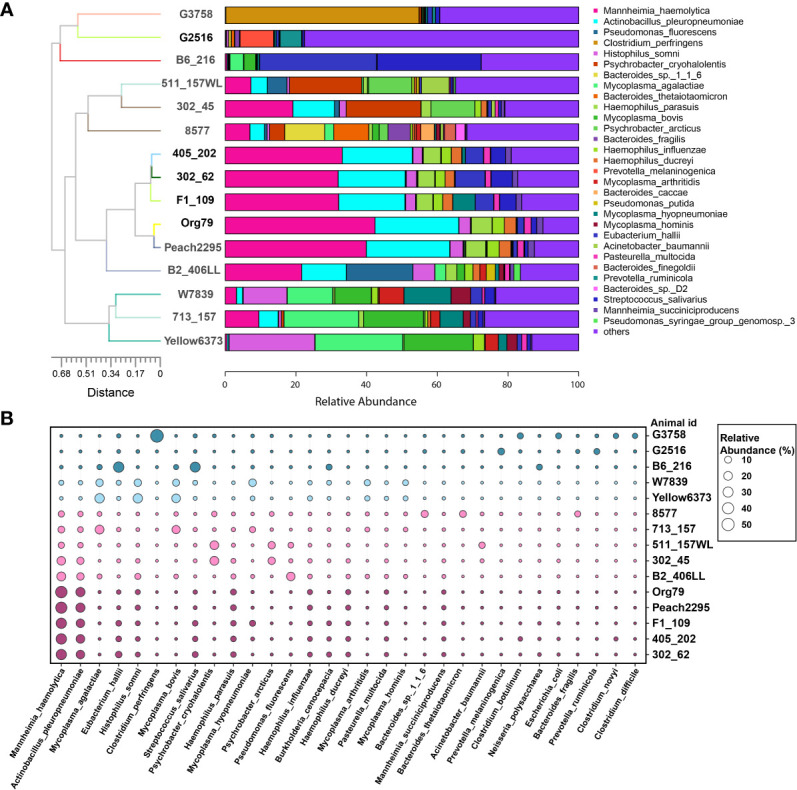
Individual variations and clusters of bovine lung microbiome. **(A)** Top 30 bacterial species in bronchoalveolar lavage were used for clustering analysis. **(B)** The major bacterial species distribution in the lungs. Circle size proportionally represents the relative abundance of bacterial species at a certain animal.

In the BAL microbial samples collected from BRD calves, through phylum and genus bar plots (**Supplementary Figure S7**), we did observe a distinct individual variation of the bovine lung microbiome. Regarding similar microbial composition found in part of the subjects, clustering analysis was performed at the species level (**Figure 8A**). Four clusters were observed among 15 BAL samples. Cluster 1, including animal IDs Yellow6373, 713_157, and W7839, mainly consisted of *Mycoplasma bovis*, *Histophilus somni*, and *Mycoplasma agalactiae* (**Figure 8B**). Cluster 2 (animal IDs B2_406LL, Peach2295, Org79, F1_109, 302_62, and 405_202) was abundant with *Mannheimia haemolytica* and *Actinobacillus pleuropneumoniae*. Cluster 3 (animal IDs 8577, 302_45, and 511_157WL) was dominated by *Mannheimia haemolytica* and *Psychrobacter cryohalolentis*. However, cluster 4 (animal IDs B6_216, G2516, and G3758) did not have a good pattern. The B6_216 had greater abundance of *Eubacterium hallii* and *Streptococcus salivarius*, while G3758 was dominated *by Clostridium perfringens*.

## Discussion

The investigation of bovine respiratory microbiome and its function benefits us in order to understand microbial functions in health and disease. Although 16S rRNA sequencing technology has been broadly used, less metagenomics of bovine respiratory microbiome is reported. This study characterized microbial communities using metagenomics in the nasopharynx and lungs of calves from worldwide geographic locations, which provides evidence that geography and niches are important factors to impact the bovine respiratory microbiome and its function. Beyond this, long-distance transportation had greater impacts on the nasopharyngeal microbiota, especially BRD pathogens, than short-distance transportation. Consistent changes in microbiota influenced by transportation were found across samples from different countries. Metagenomics results from both healthy and diseased calves suggested the associations between bovine respiratory microbiome and BRD.

Calves in different geographic locations experienced huge variations in living environment, such as breed, gender, feeding strategy, diet, altitude, climate, *etc*. These factors together result in the variation of the microbial composition in the nasopharynx across different cities or countries. In this study, although the calves were of similar age and maintained healthy throughout the trial, calves in Canada were Hereford-crossed females that had access to water and brome-alfalfa hay, while calves in China were Simmental males that were restricted from eating and drinking. It is not surprising that the diversity, structure, and composition of the nasopharyngeal microbiota in calves from different geographic locations showed distinct variations. A previous report found that weaned calves that consumed selenium-biofortified alfalfa hay for 9 weeks resulted in favorably reformed microbial communities in the nostrils (Hall et al., 2017). Differences of nasopharyngeal microbiota among Charolais and Angus–Hereford calves were also reported (Zeineldin et al., 2017a; Holman et al., 2017d; Zeineldin et al., 2017c). Thus, geography and breed effects resulting in differences of bovine respiratory microbiota increase the difficulties to understand BRD pathogenesis when using calves from different countries. In addition, signature microbiota in the nasopharynx for each geographic location were identified. *Burkholderia cenocepacia* (*Burkholderiaceae* family, Proteobacteria phylum) was the dominant species in the NPS of Saskatoon (Canada), while the NPS of Qiqihaer and Guangan had greater abundances of *Moraxellaceae* family (Proteobacteria phylum). *B. cenocepacia* is an important opportunistic respiratory pathogen of cystic fibrosis patients (Berlutti et al., 2008). *Moraxellaceae* is often found to be one of the most abundant genera in the upper respiratory tract of cattle (Mcmullen et al., 2019; Mcmullen et al., 2020), and one previous study found an association between *Moraxella* and the development of pneumonia and/or otitis in the early life of dairy calves (Lima et al., 2016). Moreover, co-occurrence between the species-associated BRD pathogens was observed in all nasopharyngeal communities. Therefore, although the calves for nasopharyngeal sampling that were from different geographic locations were clinically healthy during the trial, the nasopharynx could be a reservoir of the opportunistic pathogens for BRD. The effect of geographic locations was also a key factor to affect the functions of the bovine respiratory microbiome. As we have observed, the structure and the abundance of the function were different across geographic locations. Regarding the main functions at KEGG level 2, “membrane transport”, “signal transduction”, “transcription”, “cell growth and death”, and “cell motility” were found in the nasopharynx of Saskatoon, indicating the active, healthy, and balanced respiratory ecosystem. The calves in Saskatoon were kept healthy during the trial, which implied that the microbial community had more interactions with the host. It is not surprising that more cell growth and death and nutrient metabolism were found in the calves of Saskatoon when compared with the calves in China that experienced long-distance transportation. Therefore, homeostasis of the respiratory microbiome plays critical roles in bovine airway health, and disease or environmental factors can lead to disequilibrium in the respiratory ecosystem.

The lung microbiome is a critical point for BRD (pneumonia). In this study, the lung microbiome and its functions showed a significantly different composition and structure compared with nasopharyngeal communities regardless of their geographic locations. The BAL samples were collected at necropsy from 15 feedlot cattle that were confirmed to have died of BRD. Higher abundances of *Mannheimia haemolytica*, *Mycoplasma bovis*, and *Histophilus somni* were observed, which is in agreement with previous studies that these bacteria are BRD-associated pathogens (Rice et al., 2007; Singh et al., 2011; Nicola et al., 2017). Notably, *Mycoplasma* species (*M. agalactiae*, *M. bovis*, *M. arthritidis*, *M. hyopneumoniae*, and *M. hominis*) inter-connected with each other were observed in BRD lungs, which reflects the cooperation of pathogens to cause BRD. Based on the hypothesis that the nasopharynx is a reservoir of the opportunistic pathogens for BRD, we assumed that *Mycoplasma* species may come from the nasopharynx. A study reported that the best recovery condition for *Mycoplasma bovis* is at 36.91°C (± 0.07) and pH = 7.13 (± 0.05) under an aerobic environment (Parker et al., 2016). Bovine nasopharynx is the niche with pH of about 7 and temperature of about 37°C, which is good for the colonization and growth of *Mycoplasma* species (Chai et al., 2022). Therefore, the opportunistic pathogens for BRD may colonize the nasopharynx and disperse in and infect the lungs, which provides a new way to explore BRD pathogenesis and prevent BRD. In addition, the interactions of *Mycoplasma* species might be another reason causing BRD, which need to be investigated in future research.

BRD, also known as “shipping fever”, is caused by various stressors (*i*.*e*., weaning, transportation) that also influence the bovine respiratory microbiota (Chai et al., 2022). Understanding the association between transportation and microbial pathogen proliferation may explain BRD pathogenesis. A previous study demonstrated that transportation to a feedlot caused an abrupt shift in the nasopharyngeal microbiota of cattle using 16S rRNA sequencing, and major differences were driven mostly by *Mycoplasma* (Holman et al., 2017d). A metagenomic study confirmed that stress from long-distance shipping influences the nasopharyngeal microbiota (Cui et al., 2021). Similarly, in our study, long-distance transportation had bigger impacts on the bovine respiratory microbiota, and *Mycoplasma* species, including *M. bovis*, *M. conjunctivae*, *M. pulmonis*, and *M. hyorhinis*, increased the abundances after feedlot arrival. Thus, stressors (*i*.*e*., long-distance transportation) and the feedlot environment may provide conditions that allow for the proliferation of *Mycoplasma* in the nasopharynx. In addition, abundance of *Mycoplasma agalactiae* by both short- and long-distance transportation was observed, which gives more evidence that transportation can cause changes in *Mycoplasma* species (Stroebel et al., 2018). It was reported that stress can exacerbate respiratory hyperreactivity and inflammation in an animal model of allergic bronchial asthma (Joachim et al., 2003). Newly weaned calves experience huge stress during transportation or shipping, especially for a long distance (Zulkifli et al., 2019; Malmuthuge et al., 2021), which may cause unclear changes in the airway for *Mycoplasma* proliferation. Keeping microbial balances in bovine airway during transportation might be an alternative strategy to decrease BRD morbidity.

BRD is usually diagnosed in cattle within 4 weeks after having been transported to a feedlot (Currin and Whittier, 2005). Understanding of the temporal dynamics of the bovine respiratory microbiome from the time since arrival to the feedlot can allow us to recognize the association between microbiota and BRD. Here we found that genera associated with BRD, such as *Mycoplasma* and *Mannheimia*, were increased at a specific time after weaning and transportation. Since all of the calves were healthy and had never been diagnosed with BRD in this study, these increased pathogens might be due to the time after feedlot arrival or changes of the respiratory health status. Moreover, *Moraxella* was higher in calves that stayed with their dams. *Moraxella* is often found to be one of the most abundant genera in the upper respiratory tract of healthy cattle (Zeineldin et al., 2017a; Mcmullen et al., 2019). Therefore, *Mycoplasma* and *Mannheimia* may reach a specific level to cause clinical BRD signs, and a greater abundance of *Moraxella* in the nasopharynx might resist BRD.

The inter-individual variability of the respiratory microbiota was found in cattle and humans (Dickson et al., 2015; Holman et al., 2015b; Zeineldin et al., 2017a), which is not surprising due to individual variation. The bacterial clusters and their association with clinical signs were investigated in human respiratory microbiome studies (Zhou et al., 2019; Widder et al., 2022). However, in cattle, there were no studies to characterize the community type of the respiratory microbiota. In this study, microbial clusters of BRD lungs were found, although there was significant inter-individual variability—for instance, bacterial pathogens, including *Mycoplasma bovis*, *Histophilus somni*, and *Mycoplasma agalactiae*, were enriched in cluster 1, while *Mannheimia haemolytica* plus different pathogens formed a different cluster in the BRD lung microbiome. Previous studies found that *Mycoplasma bovis* co-infection with other respiratory bacteria (*M. haemolytica* and *H. somni*) can lead to severe pneumonic lesions, and *Mannheimia haemolytica* might be associated with *Actinobacillus pleuropneumoniae* in pigs (Gagea et al., 2006; Radaelli et al., 2008). Although the cooperation of different pathogens causing BRD is still unclear, this study provides insights that pathogenic symbiosis is one of the important causes of respiratory diseases. In future BRD studies, large-scale sampling is needed for characterizing the community type of the lung microbiome, which may reveal the interactions of bacterial pathogens and provide an alternative therapeutic strategy for BRD.

A limitation of this study may be the small sample size of lung samples. However, the dominant bacterial pathogens in each cluster could explain the subtypes of BRD pathogenesis since these samples were collected from the lungs of calves that died from BRD. The strengths of this study are the analysis of bovine nasopharyngeal microbiome from different geographic locations using metagenomics and the finding that the general pathogens were influenced by transportation regardless of geography. Future studies to assess more geographic locations of bovine respiratory microbiome and use omics to explore the interactions of the airway microbiome and the host are urgently needed.

## Conclusions

Metagenomic sequencing offers the chance to illustrate the species level of community structure and functional profile of the bovine respiratory microbiome. The geographic locations affect the composition and the functions of the bovine respiratory microbiome, and the microbiota from different sampling niches were also distinct. Additionally, transportation is an important factor driving the bovine respiratory microbiome and diseases, and long-distance transportation had a bigger chance to cause increased abundances of pathogens in the nasopharynx. Furthermore, we found that the cluster of lung microbiota from different BRD calves might provide an opportunity to come up with a new guidebook for the category of BRD pathogenesis.

## Data availability statement

The datasets presented in this study can be found in online repositories. The names of the repository/repositories and accession number(s) can be found in the article/**Supplementary Material**.

## Ethics statement

All data related to animals were downloaded from the published NCBI database.

## Author contributions

JC contributed to data collection, analysis and interpretation, and figure organization and drafted the manuscript. XL and HU contributed to draft proofing. FD contributed to data collection. JZ and YL contributed to conception, manuscript proofing, and project supervision. All authors contributed to the article and approved the submitted version.

## Funding

This project was supported by the Agriculture and Food Research Initiative competitive grant no. 20196701629869 from the USDA National Institute of Food and Agriculture, National Natural Science Foundation of China (no. 32170430), Guangdong Provincial Key Laboratory of Animal Molecular Design and Precise Breeding (2019B030301010), and Key Laboratory of Animal Molecular Design and Precise Breeding of Guangdong Higher Education Institutes (2019KSYS011).

## Conflict of interest

The authors declare that the research was conducted in the absence of any commercial or financial relationships that could be construed as a potential conflict of interest.

## Publisher’s note

All claims expressed in this article are solely those of the authors and do not necessarily represent those of their affiliated organizations, or those of the publisher, the editors and the reviewers. Any product that may be evaluated in this article, or claim that may be made by its manufacturer, is not guaranteed or endorsed by the publisher.
