# Supplementary material for: Geography, niches, and transportation influence bovine respiratory microbiome and health
Source: Front Cell Infect Microbiol. 2022 Sep 12;12:961644. doi: 10.3389/fcimb.2022.961644 (PMC9510686; doi:10.3389/fcimb.2022.961644)

Table S1 Summary of the publicly available metagenomic datasets of bovine respiratory microbiota.

| **Study title** | **Descriptions** | **Reference** |
| --- | --- | --- |
| Effect of maternal separation and transportation stress on the bovine upper respiratory tract microbiome and the immune response to resident opportunistic pathogens | A total of 112 nasopharyngeal swabs (NPS) were collected from female, healthy Hereford-cross calves in Saskatoon, Canada.  Samples were sequenced using Illumina HiSeq4000 PE100, and data accession number is PRJNA687519. | Malmuthuge et al. |
| Metagenomics Reveals That Proper Placement After Long-Distance Transportation Significantly Affects Calf Nasopharyngeal Microbiota and Is Critical for the Prevention of Respiratory Diseases | A total of 18 nasopharyngeal swabs (NPS) were collected from male, healthy Simmental calves in Qiqihaer and Guangan, China.  Samples were sequenced using Illumina HiSeq 2500 platform, and data accession number is PRJNA724913. | Cui et al. |
| Lower Respiratory Tract Microbiome and Resistome of Bovine Respiratory Disease Mortalities | A total of 12 bronchoalveolar lavage (BAL) samples were collected from feedlot calves which died from BRD in Alberta, Canada.  Samples were sequenced using Illumina HiSeq 2500 platform, and data accession number is PRJNA395911. | Klima et al. |

Figure S1 The domain level of bovine respiratory microbiome among 4 cities


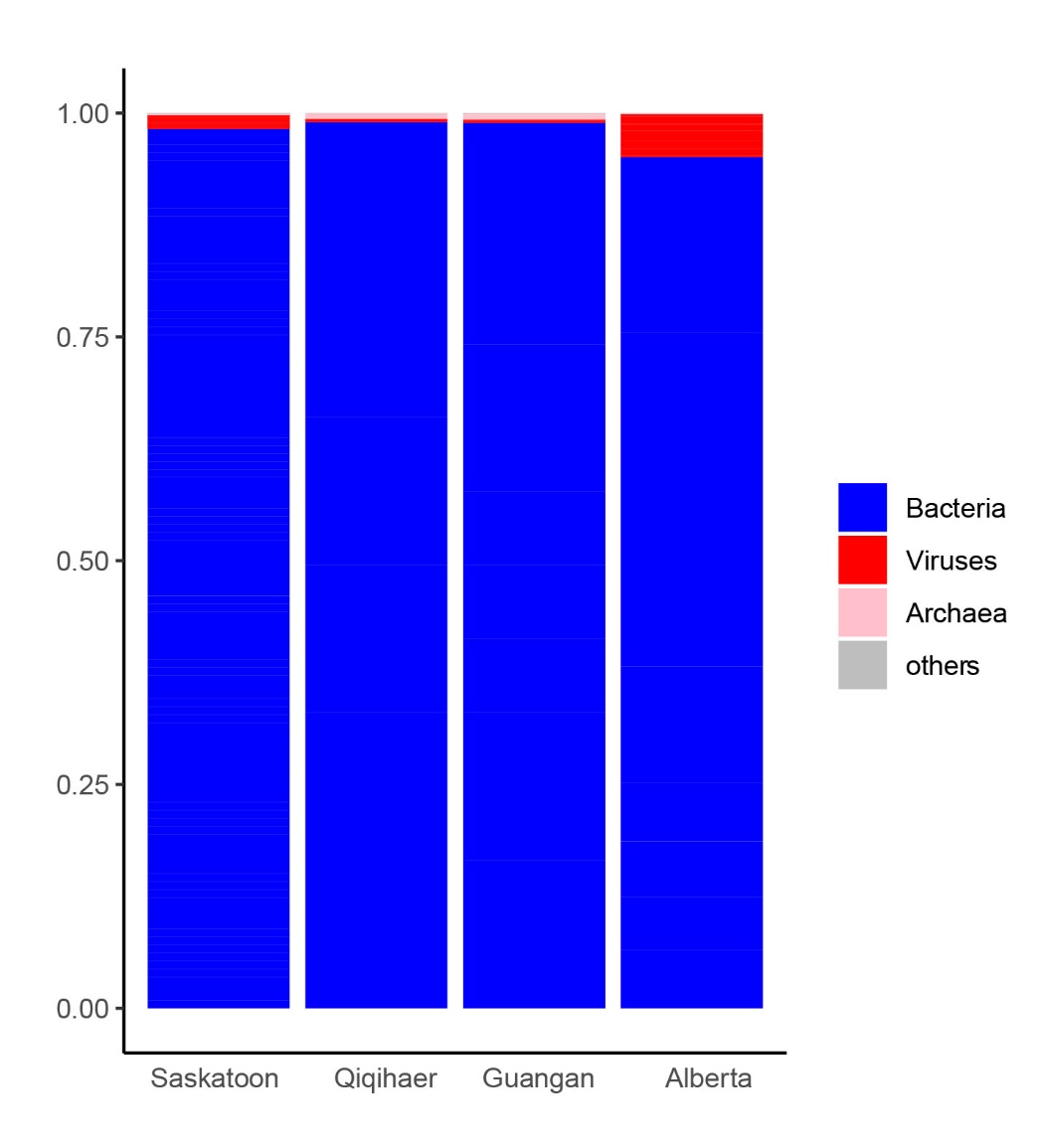


Figure S2 Alpha and beta diversities and genus composition of bovine respiratory microbiome among 4 cities


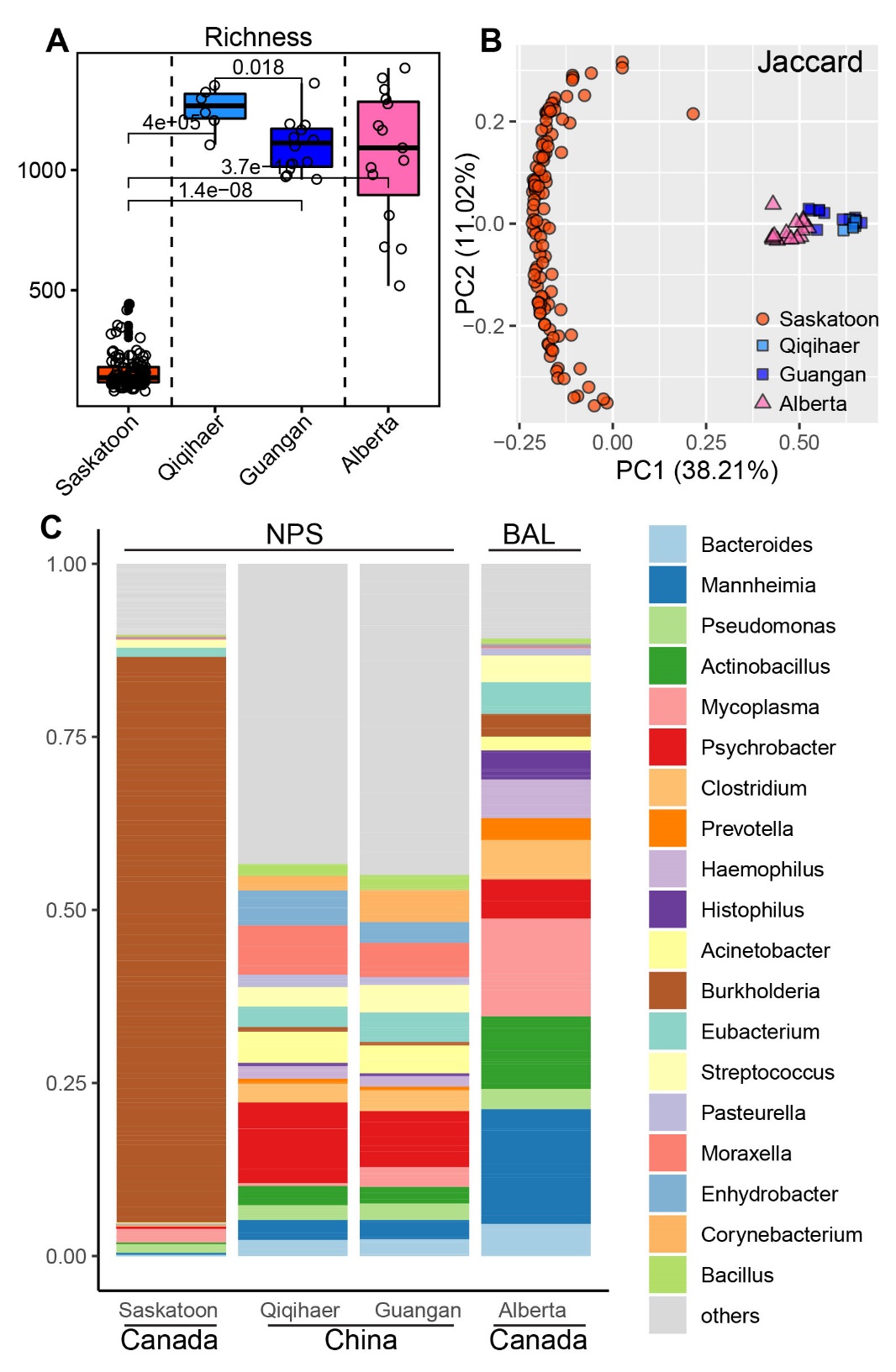


Figure S3 KEGG level 1 bovine respiratory microbiome among 4 cities


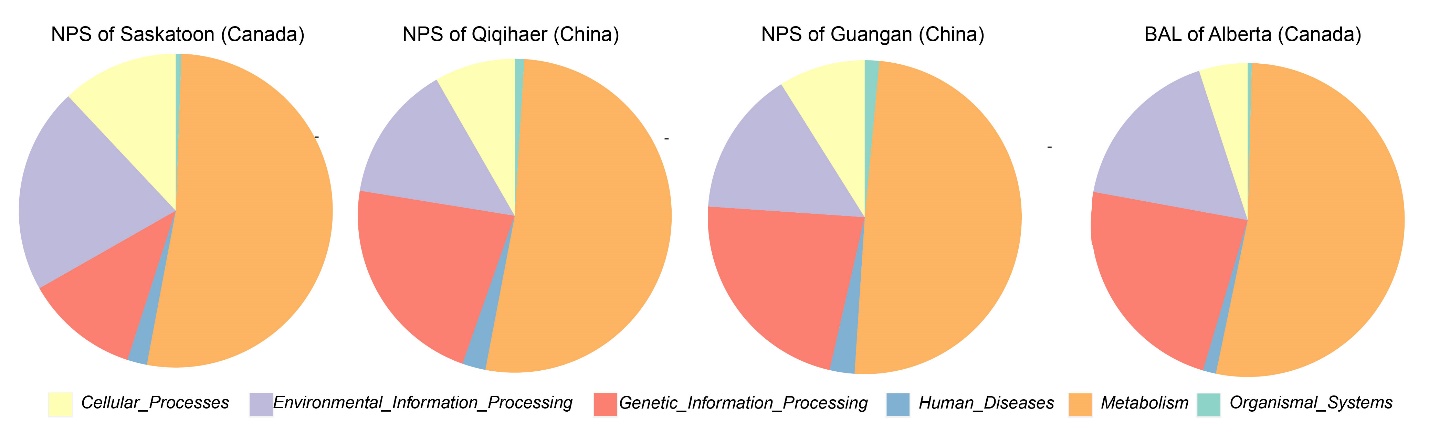


Figure S4 Signature microbial species at the time before loading to truck (BFload), unloading (Unload), and 7 days after placement and adaptive feeding (ADfeed) for Short- and Long-distance transportation identified by LEfSe


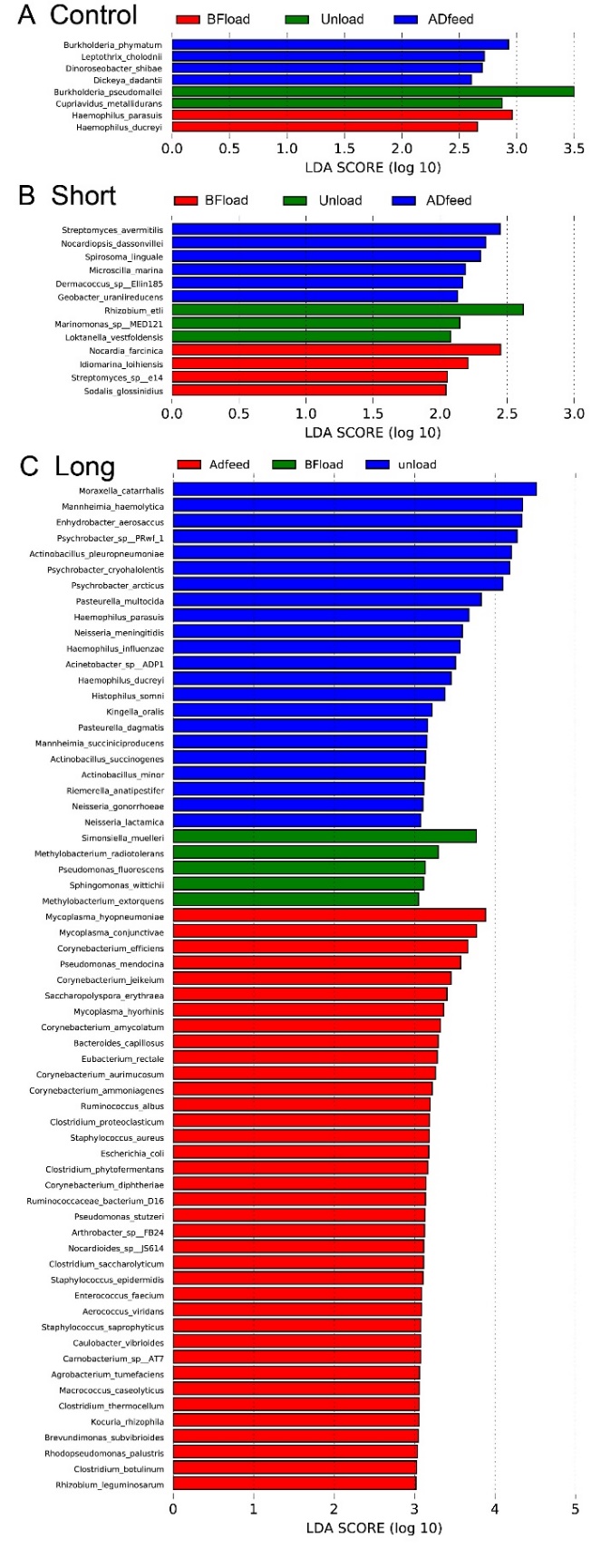


Fig S5 Alpha and beta diversities for weaning + transportation (WT) compared to control group that stayed with their dams (suckling)


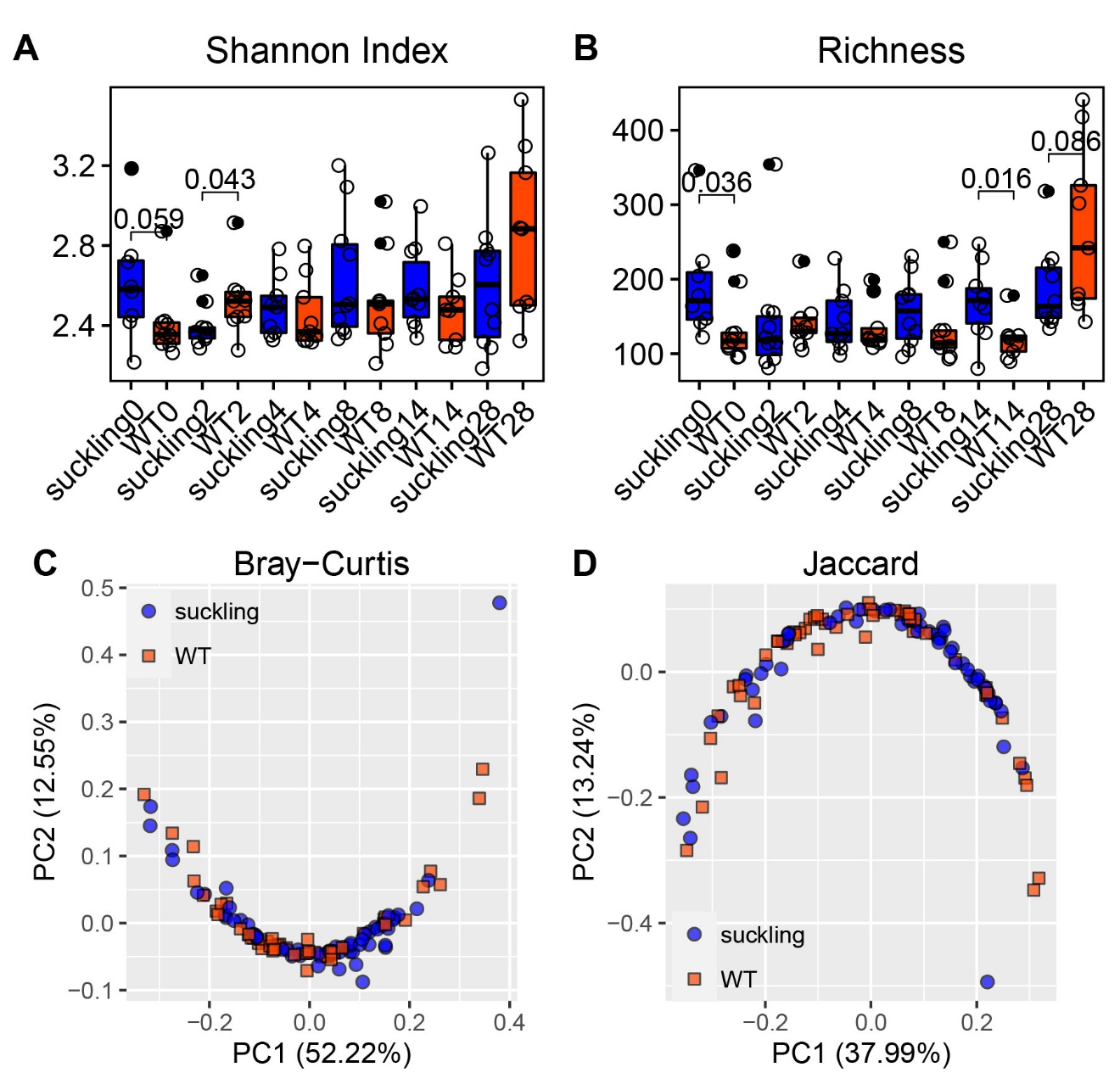


Figure S6 The temporal dynamics of alpha and beta diversities for weaning + transportation (WT) compared to control group that stayed with their dams (suckling)


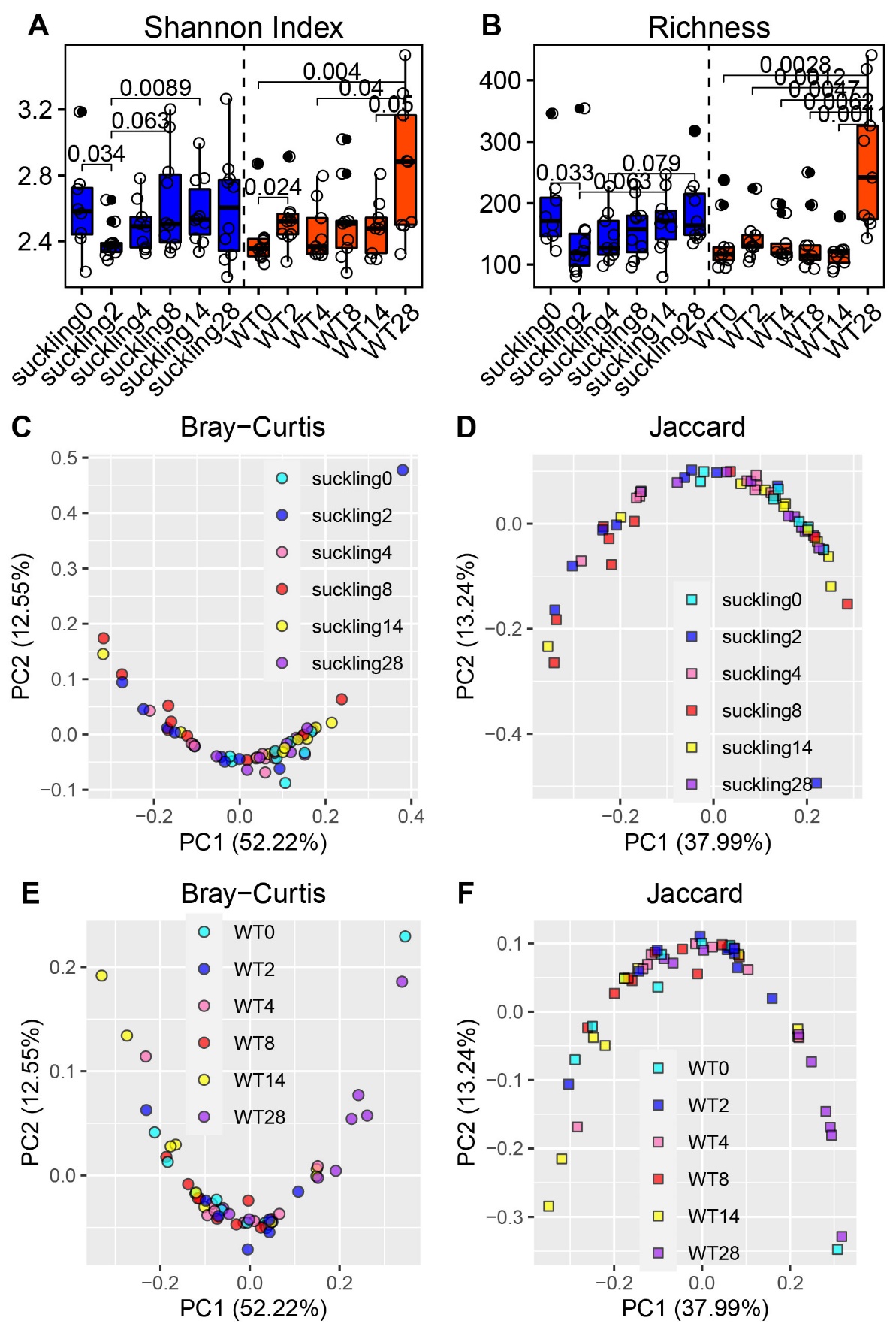


Figure S7 Phylum and genus composition of bovine lungs


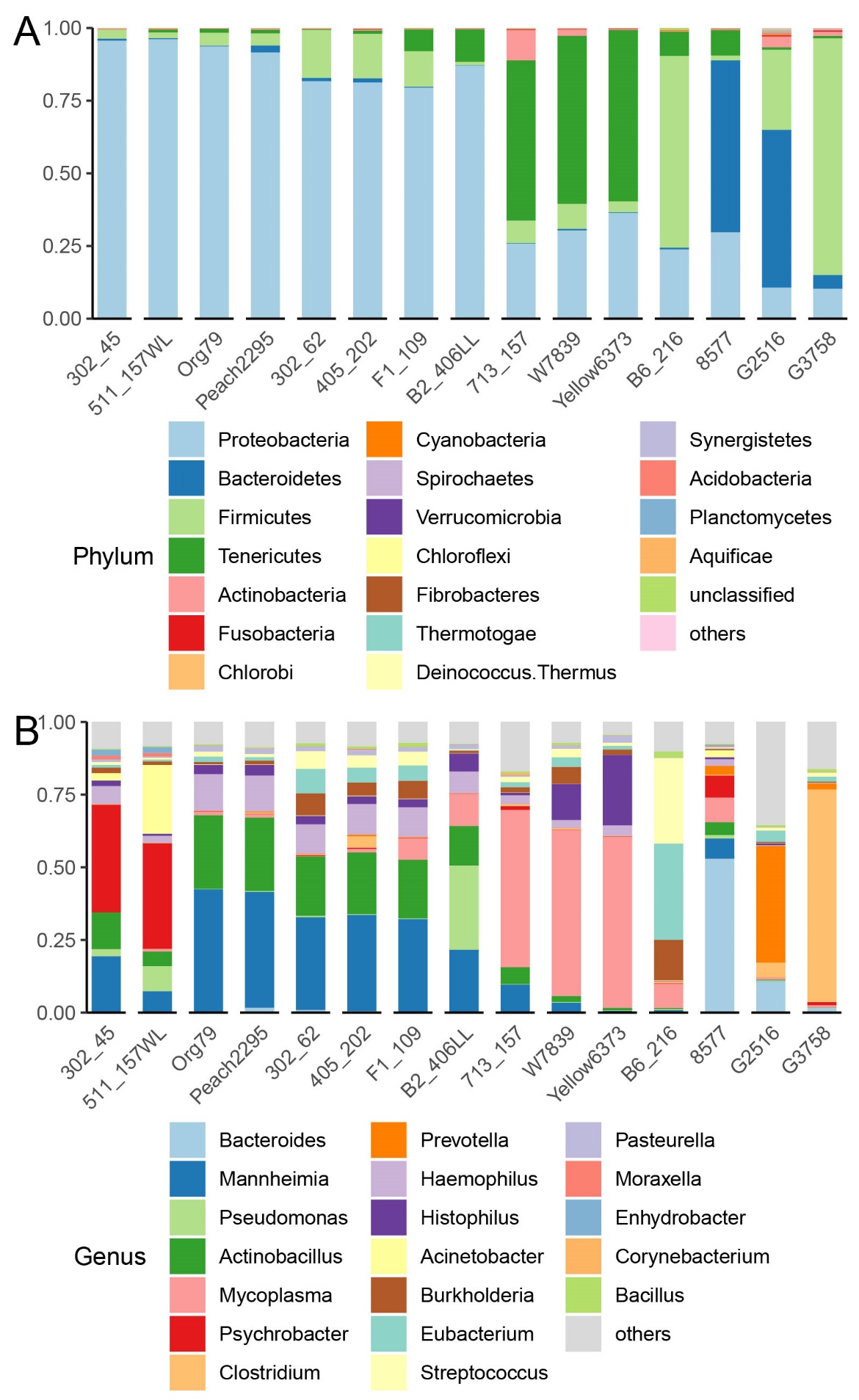

Supplement: Supplementary file 1 [file DataSheet_1.docx]
